# How female community health workers navigate work challenges and why there are still gaps in their performance: a look at female community health workers in maternal and child health in two Indian districts through a reciprocal determinism framework

**DOI:** 10.1186/s12960-017-0222-3

**Published:** 2017-07-01

**Authors:** Enisha Sarin, Sarah Smith Lunsford

**Affiliations:** 1 University Research Co., LLC, B7, 1st floor, Suncity, sector 54, Gurgaon, Haryana 122001 India; 2 EnCompass LLC, Rockville, United States of America

**Keywords:** Community health worker, Health worker performance, Social cognitive theory, Reproductive and maternal health, Gender norms, India

## Abstract

**Background:**

Accredited Social Health Activists (ASHAs) are community health workers tasked to deliver health prevention in communities and link them with the health care sector. This paper examines the social, cultural, and institutional influences that either facilitate or impede ASHAs’ abilities to deliver services effectively through the lens of the reciprocal determinism framework of social cognitive theory.

**Methods:**

We conducted 98 semi-structured, in-depth interviews with ASHAs (*n =* 49) and their family members (*n =* 49) in Gurdaspur and Mewat districts. Data were analyzed by comparing and contrasting codes leading to the identification of patterns which were explained with the help of a theoretical framework.

**Results:**

We found that while the work of ASHAs led to some positive health changes in the community, thus providing them with a sense of self-worth and motivation, community norms and beliefs as well as health system attitudes and practices limited their capacity as community health workers.

**Conclusion:**

We outline potential mechanisms for improving ASHA capacity such as improved sensitization about religious, cultural, and gender norms; enhanced communication skills; and sensitization and advocating their work with health and state officials.

## Background

### ASHA programme in India

India’s Accredited Social Health Activist (ASHA) programme was launched by the National Rural Health Mission (NRHM) (now known as the National Health Mission (NHM)) in 2005, in line with its policy of community engagement to ensure people’s participation in health.

One ASHA is responsible for conducting health promotion activities for 1000 people in a village. ASHAs are recruited from the community based on leadership and communication skills and have at minimum an eighth grade education. This education requirement is relaxed in areas where women are unqualified. Women between 25 and 45 years are preferred. The responsibilities of ASHAs include functioning as a “health care facilitator, service provider, and health activist” [1].

ASHAs’ activities in reproductive, maternal, neonatal, and child health (RMNCH) include motivating and escorting women to access antenatal care (ANC) and facility-based delivery, providing post-natal care, promoting and facilitating use of birth spacing methods, immunizations, and counseling about pregnancy-related issues including anemia management. Additionally, ASHAs distribute iron tablets, sanitary napkins, contraceptives, and pregnancy kits. They also maintain pregnancy registration records and hold village-level health meetings [1].

### CHWs and performance-related barriers in the global literature

CHW programmes, globally, have been shown to be effective in certain areas of maternal and child health including promotion of uptake of breastfeeding and immunization, essential newborn care, health education, and reduction in child morbidity and mortality [2, 3]. Home visits for neonatal care by CHWs reduce infant and neonatal deaths and stillbirths in resource-limited settings [4, 5]. Despite this, there are barriers to CHW performance.

A Cochrane Review identified organizational, social, and interpersonal factors that either facilitated or impeded CHW programmes [6]. While community acceptance of CHWs and organizational support were important for the success of CHW programmes, barriers to CHW programme success were related to the relationship with beneficiaries and the health system, prevailing socio-cultural conditions, and institutional factors. Notably, socio-cultural norms that restrict movement of female CHWs and govern acceptable male-female communications have been identified as a barrier to doing their jobs successfully [7–10]. Interpersonal barriers include fear of blame if interventions were unsuccessful, inability to meet expected needs of the community, and lack of understanding of benefits that prevented community members from using contraception [11] or having a facility-based delivery [12]. Institutional barriers include limited supplies [8, 13, 14], excessive paper work [15], and limited support from a rigid and hierarchical health system [16–18].

While the literature on obstacles CHWs face is extensive, their responses to these obstacles have not been scrutinized with the exception of several studies. CHW communication skills play an important role in overcoming barriers related to community perceptions and beliefs [19–23]. In India, female community-based workers in an urban slum were found to negotiate their relationship with other women by emphasizing common experiences, characteristics, and forming friendships [20]. In India, an evaluation of the ASHA programme in 16 states highlighted gaps in technical skills of ASHAs, and their inability to cover marginalized populations [24]. Although context-specific barriers have been identified in studies of ASHAs [17, 18, 25] that may explain why their performance is still low, what remains to be known is whether ASHAs can appropriately address these barriers and the strategies they employ to resolve these and perform as health workers.

The current study examines the influences of ASHA workers’ environments on their role as health workers and how they respond to these in the course of performing their duties. We have based our conceptual framework on Bandura’s reciprocal determinism which posits that people’s actions are determined by goals, self-efficacy, outcome expectations, and perceived facilitators, and social and structural impediments [26]. Self-efficacy is an individual’s belief in one’s ability to succeed in a specific task or situation. It is important for behavior change as it provides motivation to overcome barriers and evokes feelings of empowerment to enact change [27]. In explaining worker motivation, Franco and others developed a conceptual framework, from which we additionally borrowed, which lays down internal factors such as self-concept, external factors such as cultural influences, as well as organizational systems and structures that determine worker motivation [28]. In this framework, culture and community influence internal motivational factors such that an individual frames her actions against what is possible and expected, and the consequences of going against cultural norms [29]. Furthermore, organizational support structures and processes influence a worker’s ability to perform [28].

## Methods

### Study setting

The study was conducted in Mewat and Gurdaspur districts of Haryana and Punjab. These districts were purposively selected for their socio-economic and religious diversity. Both study districts are traditional, rural communities with prevalent unequal gender norms and religious and cultural taboos. Around 70% of Mewat’s population is Muslim [30], while Gurdaspur is predominantly Sikh. Illiteracy among currently married women is 21% in Gurdaspur [31] whereas it is 60% in Mewat [32]. Gurdaspur has a low child sex ratio (895 girls per 1000 boys) due to a cultural preference for male children. Mewat has low rates of institutional delivery (40.3%), ANC coverage (30.5%), and full immunization (20.8%) due to multiple systemic and socio-cultural factors that hinder utilization [32].

### Sampling and recruitment

One sub-center from each primary health center in an administrative block of the districts was selected to ensure representation of religious and caste groups. Within each sub-center, ASHAs who were married and consented to participate were randomly selected. In Mewat, we interviewed 9 Muslim and 15 Hindu ASHAs; in Gurdaspur District, we interviewed 21 Sikh, 1 Hindu, and 3 Christian ASHAs. We also interviewed the ASHAs’ husbands or mothers-in-law as these were primary decision makers who had influence over ASHAs’ personal and professional lives. The total sample included 49 ASHAs and 49 family members.

### Data collection procedures

Interviews were conducted using a semi-structured guide developed in English by the two authors and translated into Hindi and Punjabi by a translator. Questions were formed around the major objective of the study, i.e., the influence of family, community, and the health system on ASHA performance. Specifically, the questions pertained to (1) experience as ASHA—changes in personal life, self-perception, mobility, motivation; (2) family and community support; (3) health system support; and (4) perception of impact of work in the community. Data collection was carried out by hired qualitative data collectors—two female and one male fluent in the local languages. They received a 2-day training on qualitative interviewing techniques, data collection tools, and human subjects’ research ethics. They were supervised by the first author.

Noting cultural preference as well as the comfort level of participants, interviews with husbands were conducted by the male interviewer and ASHAs and mothers-in-law were interviewed by female interviewers. Prior to conducting the interviews, interviewers explained the study including risks and benefits of participation, and obtained written consent. Participation was voluntary. Interviews were conducted in a private location selected by ASHAs and family members and were audio-recorded with the participants’ permission. Interviewers took notes to clarify local phrases and words in the recorded interviews, which were supplemented with interviewers’ reflexive field notes prepared following each interview. Interview duration was between 45 and 90 min. Interviews were transcribed and translated into English and uploaded into the qualitative research software, Atlas.ti©, for coding and analysis. As regards data saturation, we set out to sample the requisite number of interviews as we wanted a wide representation across caste and religion. We coded and analyzed all data as each interview contributed a unique flavor to the whole.

### Ethical review

The protocol, interview guides, and consent forms were approved by the Institutional Review Boards at the University Research Co., LLC in Maryland, USA, and Center for Media Studies in New Delhi, India. Permission was sought from district health officials. Respondents were not compensated for their participation.

### Data analysis and interpretation

The two authors independently coded 15 transcripts with an initial coding list based on the topics in the interview guide. This list was revised with new and emerging codes from the data [33, 34]. New codes were then applied to the interview texts by the two authors who held weekly phone calls to discuss the coding scheme and initial findings. As one of the authors is an American female based in Washington DC and the other an Indian female based in Delhi, divergent perspectives helped in questioning and clarifying data interpretation. We stratified the analysis by the two geographic regions to explore differences in the ASHAs’ responses. However, we found that the responses of both the ASHAs and their family members were similar across settings.

As the influence of the ASHAs’ environment was examined more closely, our findings began to take shape around common themes of enabling and impeding factors that marked ASHAs’ responses and strategies. Emerging relationships between codes were explored in analytic memos. These were later verified against further codes and based on discussion between the authors. Although we did not initially start with a conceptual framework, as we worked through the analysis, it became clear that our findings could be understood through the lens of reciprocal determinism principle of social cognitive theory (SCT). We saw that the respondents talked significantly about their work identity and how that lent them social prestige and respect as well as support from the family which motivated them to work further. They also talked about “convincing” (hindi-manana para) people in their community to practice healthy behaviors and to access antenatal, facility delivery, and post natal services and how their ability to “convince” occasionally faltered in the face of socio-cultural norms and health system challenges. Thus our findings began to be empirically grounded in a new context that helped us explain patterns of reciprocity (personal and environmental factors influencing cognition and behavior of ASHA and in turn being influenced by her behavior) we saw in the narratives and to find correspondence with the theoretical framework [34, 35].

## Results

**Fig. 1:**
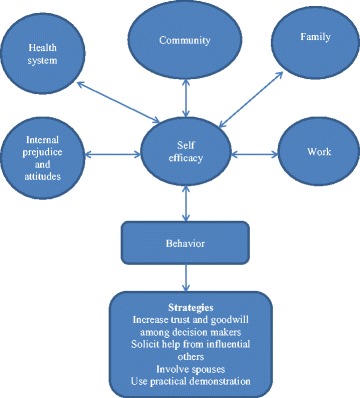
Reciprocal determinism of environmental and cognitive factors on ASHAs’ actions

The figure represents the bidirectional influence of the environment (family, community, health system) and personal factors on self-efficacy that determines ASHAs’ behavior and the actions or strategies that she undertakes (Fig. 1). These factors can act as both impediments and facilitators. We present, at first, enabling factors that strengthen an ASHA’s self-concept and self-efficacy. Subsequently, we present challenging or impeding factors to her ability to perform. The last section deals with “how” she responds to these challenges in varying ways—some of these being successful, some not.

### Enabling factors

ASHAs gain self-efficacy through doing the job and the feedback/support they get from community and family. As they gain more self-efficacy, they become better at their jobs and deliver better services to the community which results in more positive feedback and more self-efficacy.

#### Work-related enablers

By joining the workforce, ASHAs began accruing resources including financial incentives, technical knowledge, and social capital such as respect and support from family and community. These, in turn, increased an ASHA’s self-efficacy to successfully perform her duties.

Acquisition and sharing of health knowledge was one of the biggest changes that ASHAs reported before and after joining work. Providing health information earned them respect from the community, which motivated them to work:

I have become much confident now. I can talk to anybody. People seek advice from me. I have knowledge about health, so I feel confident. I want to do more work. (ASHA #44, Gurdaspur)

In addition, ASHAs attributed freedom of movement and ability to communicate with people to their sense of self-confidence.

Prior to being an ASHA I didn’t go anywhere alone. But now I can move about freely. I accompany my patients to facilities. After being ASHA my confidence has increased. (ASHA #46, Gurdaspur)

ASHAs also reported they gained an identity separate from that of their husbands and fathers and were addressed as *behenji* (respectful term for sister) and not as somebody’s wife or daughter—a recognition by the community of their professional status.

Earlier I used to feel very scared to go out and talk to people. I used to stay behind the veil. But today I can converse with anyone. While talking to the elders I do stay veiled, but I am more confident and more vocal now. (ASHA #20, Mewat)

#### Family and community enablers

Support from their family in terms of sharing household chores enabled ASHAs to do their work without hindrance as one ASHA reported, “When I go for training, my family members do all the work. This is help…sometimes I have to go for ASHA duty when I am working in the fields, then I go and my family takes up the work.” (ASHA#8, Mewat).

ASHAs reported that the community’s response in following their advice, the respect they received, the changes they saw in health practices, and their ability to apply their technical skills motivated them and increased their self-efficacy.

Many home deliveries used to take place in my village where children died during birth but now at least I am able to call ambulance and avoid such deaths. And when they get tetanus injection, even if the child takes birth at home, he does not acquire tetanus. That is why I like this work very much. (ASHA #3, Mewat).

This quote from a mother in law reveals the extent of the respect the family has gained from the ASHA’s work

Earlier no one knew us. Now since she became an ASHA the honor and respect of the family has increased. I feel very good when people come and call her that G come, you do very good work and help us in every way. Earlier no one asked our opinion. But now everyone takes the view from our family. (Mother in law #7, Mewat).

#### Health system enabler

Although ASHAs narrated their negative experiences with health facilities, many of them also spoke of the help and support they received from their immediate supervisors,

When I was new I had to do visits, some people didn’t listen to me…so it was quite problematic…then my ANM used to help me by talking to certain females and explaining things on my behalf. Now everything is fine (ASHA#2, Mewat)

### Challenges

#### Gender, cultural, and religious norms

However, the same factors that enabled ASHAs could also throw challenges, especially at the community and health system level as well as at the level of ASHAs’ own internal attitudes and prejudices. These affect an ASHA’s self-efficacy and she may feel incapacitated to deliver services. ASHAs talked about “convincing” women in the community to access health services “I convince them by telling again and again. I tell them I have no personal benefit. If woman of home is healthy then all the members of family will be healthy.” (ASHA# 29, Gurdaspur). While trying to convince, ASHAs reported experiencing frustration as expressed by one, “When patients don’t listen to us, I get frustrated.” (ASHA#33, Gurdaspur).

ASHAs reported gender, cultural, and religious norms that inhibited their clients’ freedom to make decisions about their own health, which made it difficult for ASHAs to carry out work such as linking women with ANC and institutional delivery, and registering early pregnancy.

“They don’t go (for health services) because their husbands and in laws don’t like it…out of four parts one part is still there who don’t go” (ASHA #2, Mewat)

ASHAs reported difficulty in registering pregnant women early in their pregnancy partly because of gender norms preferring sons over daughters. Couples with a female child were reluctant to disclose second pregnancies due to taboos around expectations of a male child:

People don’t inform us about pregnancy. Most problems come in case of second baby. Because if there is a daughter firs and people want son the second time, they don’t reveal about pregnancy. (ASHA #38, Gurdaspur).

Traditional belief in home deliveries, and religious proscription against immunization, birth spacing, and contraception entailed another layer of complexity to ASHAs’ health promotion activities.

I face many problems in the village. Mother in law of some patients says, “I also gave birth to children at home; I didn’t face any problem, what is the need to go to hospital?” (ASHA#33, Gurdaspur)

In my area only two or three houses are such where females do not come for immunization because they say that God will take care Himself. They just don’t understand. (ASHA#8, Mewat)

Such problems became acute when ASHAs visited marginalized communities, such as Gujjars in Gurdaspur district,

I face more difficulty when I talk with Gujjars. They are not ready for simple blood test. They say - she is fine madam, don’t advise us. We don’t want to go to hospital. They are not ready for USG (ultrasonograph). It is very difficult to get them ready for immunization. They don’t listen to me. (ASHA# 46, Gurdaspur)

#### Internal cultural, gender, and religious beliefs

Although ASHAs took effort in convincing families to seek healthcare services, adherence to their own cultural traditions appeared to be a hindrance. For instance, Hindu ASHAs reported not going inside houses of Muslim families or eating and drinking in these households, reinforcing age-old customs impeded their ability to deliver services. Because there was limited interaction with Muslim households, Hindu ASHAs did not persist in their efforts, perceiving impediments in promoting birth spacing methods,

Mostly women are like this only among Muslims. Nobody takes contraception. I keep asking them again and again. Those that want, I give, those that don’t want, I don’t ask them. (ASHA #25, Mewat).

Furthermore, ASHAs reflected their own gender bias as expressed in self-reports of son preference. This may have implications in successfully accomplishing activities like early registration of pregnancy. Being a product of the same socio-cultural milieu, ASHAs may have internalized these social values as typified by one ASHA’s personal narrative,

…When doctors told me that there are twins…I wanted to abort. I was scared that I will get two daughters. I did not want to have two daughters. Then I gave birth to a daughter and a son. After delivery my daughter was taken care of and brought up by my parents-in-law. (ASHA # 42, Gurdaspur).

#### Health system challenges

While ASHAs aimed to link the community with the health system, their efforts were undermined when they or their patients experienced disrespect in health centers as reported by some ASHAs. ASHAs also reported that patients lost their confidence in hospitals when doctors or testing facilities were unavailable.

We explain to patients that if you deliver your child in the hospital you will get better care. Upon their request, we stay with them, even at night. But when after all this effort someone raises their voice at us in front of the patient and says keep quiet, mind your own business and do not talk useless nonsense, then it is hurtful and humiliating. (ASHA#27, Mewat)

The husband of an ASHA who accompanied her on her work had this to report about unavailability of doctors

R: Sometimes there is problem with government hospitals. Few days back, we took one delivery case to community health centre (CHC), but there was no doctor at night duty. So patient’s family got angry with us. Then they went to private hospital.

I: Then how did you feel about that?

R: We felt very bad; there is loss in patient’s trust in us.

I: Did you ask anybody about this?

R: My wife talked to ANM. I asked her to tell this to her officers. But nothing happened (Husband #44, Gurdaspur)

Another challenge was when there was a lack of test kits and other facilities in the hospitals as expressed by this ASHA,

I face problem during scanning. It is not available inside hospital and patients have to go outside. Some patients can’t afford it. (ASHA#43, Gurdaspur)

Furthermore, receiving delayed incentives was another challenge for ASHAs’ motivation to continue working,

I get my salary after 5–6 months. I am not satisfied with the salary. Many times, I wanted to leave this job, but every time my husband suggested me to continue this job. He said that something is better than nothing. (ASHA#34, Gurdaspur)

### Strategies and improvement in access to health services

To overcome the challenges described above, ASHAs took several strategies, some of which were perceived as improving utilization of ANC, institutional delivery, and immunization services. However, they reported that these strategies did not always work with all people.

#### Inter-personal relationships

Mothers-in-law, husbands, and religious leaders influenced decisions to seek health services. ASHAs sought to build their relationships with mothers-in-law and husbands to encourage pregnant woman to get care; however, these relationships needed to be built in a socio-culturally appropriate manner. ASHAs gained trust and approval of mothers-in-law by providing incentives such as free medicines.

The mother-in-law does not allow daughter-in-law to follow my advice. Then I go and explain to the mothers-in-law first. I give them medicines like paracetemol and iron tablets and make them happy first. (ASHA #27, Mewat).

Direct communication with husbands of pregnant women was not easy due to cultural norms so many ASHAs engaged their husbands in talking with other men and providing contraceptives.

I feel hesitation in talking with men. They get information from my husband as he knows a lot of things after going with me to meetings (ASHA #32, Gurdaspur)

In order not to jeopardize community values in promoting birth spacing, one ASHA advised women who approached her for contraceptives to first speak with their husbands to avoid conflict,

Some women come to me and ask for measures to stop children. I tell them convince your husband first, otherwise if you do something on your own, your husband will say unnecessary things. (ASHA #10, Mewat)

ASHAs suggested involving those who had influence in the community such as religious leaders in raising awareness about immunization:

I would say announcements should be made in mosques so that people get affected that their maulvi (religious leader) is telling them to get immunized (ASHA #7, Mewat).

ASHAs also reported using peer learning to persuade women to access health care. When the ASHA was of the same religion, she communicated about health benefits by using herself as an example, “I say that I am from the same religion…religion does not tell you that you have so many children…and you can’t even take care of them. Now women have started to understand.” (ASHA #10, Mewat).

#### Providing services to community members

ASHAs reported undertaking activities that increased their good will in the community, for example, to get buy-in from the community, they provided “services” to the community that were outside the scope of their work, such as obtaining Out-Patient Department (OPD) registration cards and accompanying women to the health center for minor illnesses.

Sometimes I go to the hospital and make patients’ OPD slip. Then I go to their homes and give them OPD slips. Just for the sake of convincing them to go to health facility, so that they don’t make any excuses. (ASHA #33, Gurdaspur).

#### Practical demonstration

ASHAs arranged for pregnant women and decision-makers to visit health facilities to help remove fears and doubts about the formal health care system, including beliefs that deliveries were conducted by male doctors and hospitals were unclean.

I take the ladies to the hospital so that they see for themselves the facilities of government hospital and hence develop trust in me. (ASHA #7, Mewat)

## Discussion

Our findings show that ASHAs’ actions are influenced by internal, cultural, community, and organizational factors [28]. We included the construct of self-efficacy that depicts the ability of ASHAs to perform their services through a reciprocal determinism perspective. When faced with work challenges, an ASHA may feel she has or does not have the capacity to overcome these, depending on her perception of members of marginalized communities and religious minority groups, her abilities, how the community saw her, and what strategies would be most successful given the socio-cultural milieu.

Reinforcers such as respect and prestige in the community, self-confidence emanating from application of knowledge, family support and support from supervisors assisted ASHAs in performing their role. Community recognition imparted a sense of self-worth among ASHAs that motivated them to work harder, echoing a study that a CHW’s motivation is found to be based on interpersonal relationships borne of trust and social standing in the community [29, 36]. In the SCT literature, self-esteem and self-efficacy are functionally dependent and influence one another. When ASHAs believed in themselves, they took creative action to promote healthy behavior such as taking families to health centers. ASHAs employed empathetic communication when talking to women. CHWs are readily accepted when close relationships are forged with the community based on trust and empathy [12, 22, 37–39]. Similarly, support from their families in household chores enabled them to undertake many activities while involvement of their husbands enabled them to indirectly influence the men in the community.

Occasionally the same community threw challenges which ASHAs attempted to deal with by undertaking certain strategies. In order to not jeopardize their social standing, an ASHA may take recourse to actions that have social approval at its core. ASHAs advised women to convince their husbands before using contraceptives reflecting the influence of community beliefs on their behavior. ASHAs did not feel it was in their control to do certain tasks such as persuading men to practice healthy behaviors or allow their wives. ASHAs utilized external resources (e.g., religious leaders, husbands) as they were deemed more influential, thus reflecting the theoretical principle that people access those who have power to act on their behalf when they were not in control of social situations [40]. Furthermore, ASHAs reported having trouble engaging marginalized people, sometimes perceiving them as impervious to health information. This finding mirrors a SCT principle that when people believe their actions cannot produce desired results, they lose the incentive to act [40]. While we cannot say definitively that ASHAs stopped activities with people from different ethnicities, their frustrations and prejudices appear to have lessened their efforts. Limited coverage of the marginalized has also been one of the main findings in the ASHA evaluation [24]. This shows that social norms exert influence over ASHAs’ beliefs or feelings of ability to undertake activities, thus extending the explanation of CHW motivation, retention, and performance which generally focuses more on individual cognition tending to underestimate the impact of community influence and culture [29].

Additionally, ASHAs were demotivated when faced with issues such as lack of facilities in hospitals, poor treatment by clinic staff, and infrequent or irregular incentives. Health system obstacles have been found to impede ASHAs’ performance and morale [17, 18, 41] and have led to reviews and studies that highlight the importance of intervening on the health system level with a collaborative effort from the community [29, 42].

In the reciprocal determinism framework, a person’s beliefs may influence behavior, but an environmentally induced change in behavior can affect a change in beliefs [43]. ASHAs are bound by their own social values and therefore may not be able to communicate about gender discrimination (e.g. son preference). Hindu ASHAs were bound by cultural taboos that hindered their ability to interact freely with Muslims or other socially marginalized families which might make the ASHAs less credible with Muslim neighbors. Therefore, the framework helps in identifying and suggesting steps in improving the performance of CHWs. An environmentally induced change in behavior can occur through strengthening training curriculum, community engagement, and health system reinforcements.

### Implications for ASHA programme

There is a need for thorough sensitization about gender, religious, and cultural issues. The ASHA training curriculum includes a section on gender sensitization, designed to be taught over 3 days. Given our findings, it is not clear whether exposure to this information is sufficient to address existing gender norms in the community and self. Although there is provision for refresher courses, these are not uniformly implemented across all states. Similarly, due to the gap in coverage, a brochure on “Reaching the unreached” had been developed by the National Health Systems Resource Centre, an apex body for technical assistance set up by the Government of India. However, it is not clear whether distributing a brochure without orienting ASHAs to the content would address cultural norms and prejudices held by ASHAs. Rowe and others had pointed out the inadequacy of written guidelines for improving performance among CHW [44]. During our data collection period, the brochure was not distributed in the study sites. A 2015 evaluation of the ASHA programme [24] also pointed out the difficulties ASHAs faced in attending trainings and proposed recommendations to reiterate skills training at the monthly meetings. Although we saw these meetings being implemented in our study districts, emphasis was on technical rather than inter-personal skills. The meetings need to be strengthened and systematized with sensitization on gender and cultural issues being given equal importance. Furthermore, ASHAs need to be given a non-punitive space whereby they can share lessons learned while performing their role with their supervisors and seniors who can then help in strengthening the gaps and weaknesses by providing appropriate support. Moreover, respect and recognition from the community and support from the family is a vital factor in increasing ASHAs’ self-confidence and efficaciousness; and may be enhanced through community engagement in ASHAs’ work, a recommendation that is reiterated in reviews and studies of CHW performance [42, 45, 46]. Support of district health authorities who validate the work of ASHAs can enhance their credibility [47] while the role of supportive civil society and local political structures can also promote CHW [48].

Additionally, selection procedures of ASHAs need to be re-examined, as there are many Hindu ASHAs in predominantly Muslim areas like Mewat, where convincing women seems to be difficult. Greater organizational support in terms of available resources and facilities in hospitals, and supportive supervision are needed to assist ASHAs in tasks that they find challenging so that the good will and trust they engender in the community can continue. Incentive schemes could be responsive to the time and effort required to complete tasks and the out-of-pocket costs incurred while working as an ASHA, and to ensure timely and complete payment of incentives to ASHAs [49].

ASHAs’ prestige in the health system can be increased by implementing training of professional providers by CHWs on talking to patients with empathy, which has been widely recommended [50, 51]. There is an urgent need for promoting better interpersonal skills as a major obstacle in seeking public institutional care is lack of respect shown by health providers [52]. Lessons from ASHAs’ experiences can be one mechanism for improving patient-provider relationship [53].

There is an extensive literature on determinants of performance and motivation of CHW and while no single explanation or intervention that improves performance has been discovered, the importance of the support that the community and health system provides has been reiterated. Our study is another attempt to explore what determines CHW actions and behaviors in the Indian context but it has certain caveats.

### Limitations

While we employed the SCT framework to explain the reciprocal influence of the environment on ASHAs’ actions, we did not specifically examine the individual constructs of SCT. Neither have we measured performance of ASHAs. Additionally, we have not examined how motivation affects self-efficacy but instead have used these two terms interchangeably to point out their importance in ASHA’s performance. Our narratives hinge on these constructs without directly measuring them. Our findings do resonate with the notion of personal agency operating within socio-structural influences that lead people to be producers as well as products of social systems [40]. A further limitation is that we did not present the findings to study participants and therefore lost out on the opportunity to validate the findings.

## Conclusions

Our research found that ASHAs’ roles as social change agents influencing social determinants of health, a role that was envisioned in the conceptualization of the ASHA programme, is severely limited by pervading socio-cultural norms. Yet, as this paper identified, there are areas that can be improved in the ASHA programme, or CHW programmes in general, primarily in capacitating CHWs and creating spaces for more positive, non-punitive interaction with the health system and community.

## Abbreviations

ANC: Antenatal care
ANM: Auxiliary nurse midwife
ASHAs: Accredited Social Health Activists
CHW: Community health worker
NHM: National Health Mission
NRHM: National Rural Health Mission
